# Genome-wide association analysis provides insights into the genomics and extracellular expression of *Staphylococcus aureus* proteases

**DOI:** 10.1080/21505594.2025.2543116

**Published:** 2025-08-03

**Authors:** Shuxian Li, William Monteith, Emily Rudolph, Samuel K Sheppard, Maisem Laabei

**Affiliations:** a Department of Life Sciences, University of Bath, Bath, UK; b Ineos Oxford Institute, Department of Biology, University of Oxford, Oxford, UK; c School of Cellular and Molecular Medicine, University of Bristol, Bristol, UK

**Keywords:** *Staphylococcus aureus*, protease expression, GWAS, virulence, pathogenicity

## Abstract

Extracellular proteases are a class of *Staphylococcus aureus* virulence factors that thwart the immune system, promote nutrient acquisition, and shape the activity of virulence determinants. *S. aureus* displays considerable phenotypic and genotypic variation within clinically important lineages, giving rise to diverse infection types. Therefore, understanding how protease expression influences pathogenicity requires consideration of the underlying genes and their regulation in natural populations. In this study we determined the protease activity of 134 USA300 *S. aureus* isolates from clinical infections and asymptomatic carriage. In high-throughput casein hydrolysis assays, bloodstream infection isolates had significantly lower protease activity than carriage isolates. To identify the genetic variation underlying this variation in protease expression, we employed a *k*-mer-based genome wide association study, identifying 68 genes with polymorphisms significantly associated with proteolytic activity. Population-scale genomic variation was compared with strains from a sequenced-defined transposon library, validating the function of 27 loci that were significantly associated with decreased protease expression. Associated genes included known protease-regulating genes, including *agrA*, but most were novel. These included genes linked to central metabolism, permeases, transporters, and membrane proteins. Characterizing the complexity of protease regulation and expression will enhance our fundamental understanding of *S. aureus* virulence which may result in improved treatment options for problematic clinical *S. aureus* infections.

## Introduction

*Staphylococcus aureus* is a major human pathogen responsible for a range of diseases from mild skin infections to life-threatening illnesses such as septicaemia and endocarditis [1]. Worryingly, *S. aureus* infections are increasingly difficult to treat due to rising multi-drug resistance. Recent global estimates of bacterial antimicrobial resistance burden have indicated that deaths attributed to methicillin-resistant *S. aureus* (MRSA) have increased more than any other bacterial pathogen since 1990 [2].

A significant element in *S. aureus* pathogenicity is the highly coordinated production of several classes of virulence factors that target multiple components of host immunity. These proteins facilitate dissemination to new infection sites and elicit a variety of syndromes that can become fatal to the host [3]. One specific class of secreted virulence factors are the extracellular proteases. *S. aureus* produces 10 major extracellular proteases: a zinc metalloprotease aureolysin (*aur*); a serine V8 protease (*sspA*); two cysteine proteases (staphopain A, *scpA* and staphopain B, *sspB*); and 6 serine protease-like enzymes (*splABCDEF*) [4,5].

Secreted staphylococcal proteases can cleave a variety of host proteins including antimicrobial peptides (AMPs) such as LL37 [6], immunoglobulins [7] and complement proteins [8–10], promoting immune evasion and manipulation of central immune responses. Proteases produced by *S. aureus* participate in the degradation of extracellular matrix molecules such as fibrinogen, elastin, and collagen [11,12], which can result in tissue destruction and transmission. These proteases can also degrade staphylococcal virulence factors such as the cell-wall anchored fibrinogen-binding proteins [13] and clumping factor B [14]. This self-degradation may facilitate metastatic infection from sites of initial colonization to deeper tissue, which is mediated by surface adhesins. Interestingly, mutants devoid of all 10 secreted proteases display fitness defects when grown in peptide-rich media, indicating the role of proteases in nutrient acquisition [15]. In addition, the protease null mutant also exhibited decreased survival in human serum and blood challenge models, most likely due to the reduced capacity to withstand destruction mediated by AMPs and phagocytes that recognize complement and IgG bound bacteria [15]. While extracellular proteases are known to contribute to infection, protease null strains can exhibit hypervirulent phenotypes in murine sepsis models. This can be explained by extracellular proteases modulating the severity of infection by controlling the stability and abundance of specific virulence factors [15,16].

Protease expression is tightly regulated by a network of global virulence regulatory systems. These regulators include two component systems, Agr, ArlRS and SaeRS, the SarA protein family of DNA-binding proteins (SarA, SarS, SarR, SarV, SarZ) and transcription factors CodY, MgrA, and Rot [17,18]. In addition, using the sequence-defined Nebraska transposon mutant library (NTML) [19] several other less characterised genes have been identified that modulate protease expression [18,19].

Genotype-phenotype analysis of bacterial virulence has been instrumental in exploring the genetic basis of pathogenicity in *S. aureus*. Previous works by our group have shown significant variation in pathogenicity traits across *S. aureus* clinical isolates [20–22]. Functional genomics approaches have identified several novel loci that associated with toxin production [20–22] and biofilm formation [21] in *S. aureus*.

To broaden understanding of protease function in staphylococcal pathogenicity we screened protease expression using a previously described cohort of *S. aureus* clinical isolates derived from different infection sources [20]. Using a functional genomics approach employing phenotypic data and genome wide association studies (GWAS), we identified several novel and previously described loci associated with protease expression. Lastly using the NTML we functionally verified the role of several of these new loci in altering protease expression in *S. aureus.*

## Materials and methods

### Bacterial strains and growth conditions

The USA300 MRSA clinical isolates used in this study were isolated as described previously [20,23] and are outlined in the Supplementary Material (http://doi.org/10.6084/m9.figshare.28203137; Supplementary Table 1). *S. aureus* was grown on Tryptic Soy Agar (TSA, Sigma) and isolated colonies were cultured for 18 h at 37°C with 180 rpm shaking in 5 mL of Tryptic Soy Broth (TSB, Sigma) in a 25 mL flat-bottom glass tube. For *S. aureus* mutants containing the mariner *bursa aurealis* transposon obtained from the NTML, the transposon was maintained by supplementation with 5 µg/mL erythromycin. Bacterial supernatants were harvested from normalised bacterial cultures following 18 h growth by centrifugation at 14,000 rpm for 10 min.

### Protease activity assay

A casein hydrolysis assay was optimized based on a previously described well diffusion method [20]. Skim milk and TSA agar were autoclaved separately and mixed at 40–50°C. Petri dishes were divided into quadrants, and one well with a diameter of 8.5 mm was punctured in each quadrant. Plates were made using 25 mL of 3% casein agar to each petri dish, followed by inoculating 50 µL of 18 h supernatant into the wells and incubating in a static incubator for 24-48 h at 37°C. *S. aureus* strain LAC (AH1263) and the complete null protease mutant (AH1919), a strain where all known proteases have been disrupted or deleted were used as control strains [24]. Diameter of hydrolysis was calculated, and percentage protease activity was determined using strain AH1263 as 100% and strain AH1919 as 0%.

### Genome-wide association studies

In our previous study, we sequenced the genomes of 134 *S. aureus* USA300 clinical isolates that were isolated from with bacteraemia, skin and soft tissue infection or from asymptomatic skin or nasal patients [20]. The genomes were aligned against the genome of the reference strain FPR33757 using SNIPPY v4.6.0 on default settings (https://github.com/tseemann/snippy) and single nucleotide polymorphisms (SNPs) were identified. To compare SNP identities based on their positions, the core SNPs were concatenated utilizing SNIPPY-CORE. The “−gtr” flag was employed to disregard sites that exhibit monomorphism across all isolates and the reference strain. To minimise the impact of horizontal gene transfer on phylogenetic reconstruction, the alignment file was processed with GUBBINS v3.1.0 (https://github.com/nickjcroucher/gubbins). SNP sites were then extracted from FASTA files using SNP_SITES v2.0.0 (https://github.com/sanger-pathogens/snp-sites). The maximum likelihood phylogenetic tree was then constructed utilizing FastTree v2.1.11 (https://github.com/PavelTorgashov/FastTree) employing a generalized time-reversible model. The resulting tree was colorized and visualized using iTOL v6.6 (https://itol.embl.de/).

Whole genome assemblies were annotated using Prokka (version 1.12) [25]. Annotated assemblies were processed by the PIRATE pan-genome pipeline (version 1.0.4) [26] to identify clusters of orthologous genes [27]. Genes that were present in more than 95% of the isolates were designated as components of the core genome. Using the genome of the 134 clinical isolates, GWAS were performed to examine genetic variants associated with protease activity. To capture genetic variation, variable-length *k*-mers were extracted from the assemblies as unitigs. Pyseer v1.3.6 [28] was then employed to apply an elastic net model, examining the association between these unitigs and phenotypic traits. The lasso regression model was implemented and the default correlation filter of 25% was employed. An explicit correction was implemented by applying a genetic relatedness matrix derived from the inferred population phylogeny to adjust the *p*-values of associated variants. The phylogenetically corrected *p*-values were subsequently utilized by a suite of tools to elucidate the contribution of genetic variation highlighted by the model. For the inference of gene function, unitigs were mapped to the *S. aureus* USA300_TCH1516 complete reference genome using BWA-MEM v0.7.17.

### Statistical analysis

The graphs presented in this study were generated in GraphPad Prism 9.4.1. One-way ANOVA with Dunnett’s multiple comparison test was used to examine differences between experimental data where *p* value of < 0.05 was considered to be significant. Correlation analysis was conducted with the Spearman correlation, with the resultant correlation coefficient (r) used for analysis.

## Results

### Casein agar hydrolysis determines protease expression in S. aureus

**Figure 1.**
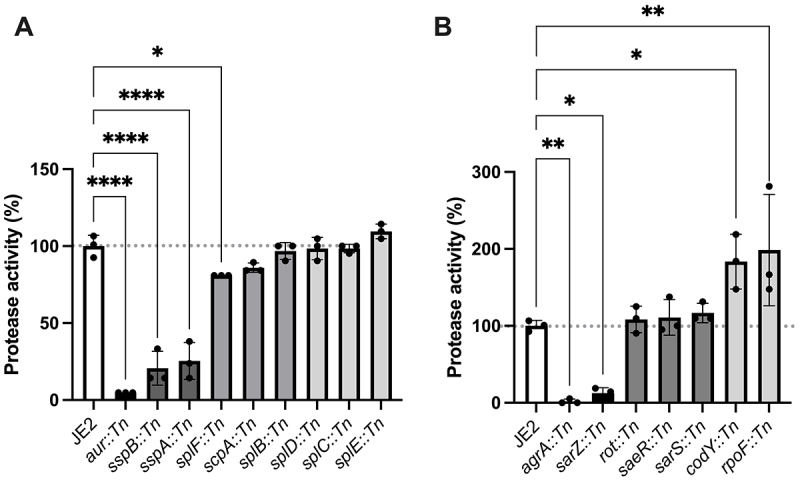
Casein hydrolysis assay comparing protease activity in selected *S. aureus* mutants. Isogenic transposon (Tn) disrupted A) protease mutants and B) selected virulence gene regulators were examined for proteolytic activity. Diameter of hydrolysis was calculated, and percentage protease activity was determined using strain LAC as 100% and the complete protease null strain as 0%. Three biological repeats were included. Each repeat is represented by dots and errors bars represent the standard deviation. Statistical differences were calculated by one-way ANOVA, *p<0.05, **p<0.01, ****p<0.0001.

The casein agar hydrolysis assay has been used to determine overall protease expression of bacterial species such as *S. aureus* [19,20], yet the exact *S. aureus* proteases that cause casein hydrolysis have not been previously reported. Using individual isogenic mutants of the known proteases we examined the individual contribution of proteases (except for *splA* as there is no *splA*::Tn mutant available in the NTML), and compared to both the wild-type and null protease mutants (Figure 1A). Tn disruption of *aur* (*p* < 0.0001), *sspA* (*p* < 0.0001), *sspB* (*p* < 0.0001), and *splF* (*p* = 0.024) had significantly reduced protease activity compared to wildtype (JE2). However, disruption of *scpA*, *splB*, *splC*, *splD* or *splE* had no impact on casein hydrolysis in our assay (Figure 1A). Given the importance of global virulence regulators in controlling protease expression we also explored the impact of Tn disruption on a subset of these regulators. Disruption of activators of protease expression, *agrA* and *sarZ*, resulted in decreased protease expression and inactivation of repressors of protease production, *codY* and *rpoF/sigB*, lead to enhanced protease activity as reported by casein hydrolysis (Figure 1B; images of casein hydrolysis assay for Figure 1A–B provided in Supplementary Figure 1). Importantly, disruption of other key regulators such as *sarS*, *rot* and *saeR* did not result in significant alteration in protease activity. Combined, protease activity data from screening Tn mutants of known secreted proteases and characterised regulators indicated that this assay can be used to report on the proteolytic activity of four major extracellular proteases and identify genes that when mutated result in decreased or enhanced proteolytic activity.

### Protease production differs among genetically related S. aureus isolates from distinct infection sources

There is significant intra- and inter-clonal variation in virulence factor expression in *S. aureus* [20–22]. Furthermore, *S. aureus* with lower levels of toxin production are associated with severe infection compared to those strains isolated from milder skin infections or asymptomatic carriage [20]. To examine the correlation between the secretion of proteases and disease severity, we performed a protease activity screen using a collection of 134 genome-sequenced clinical isolates, which correspond to a single clone (USA300, ST8, PVL+, *SCCmec* type IV MRSA), isolated from nose or skin of healthy volunteers as carriage (38 isolates), skin and soft tissue infection (SSTI) (60 isolates) or bacteraemia (36 isolates) [20,23].

**Figure 2.**
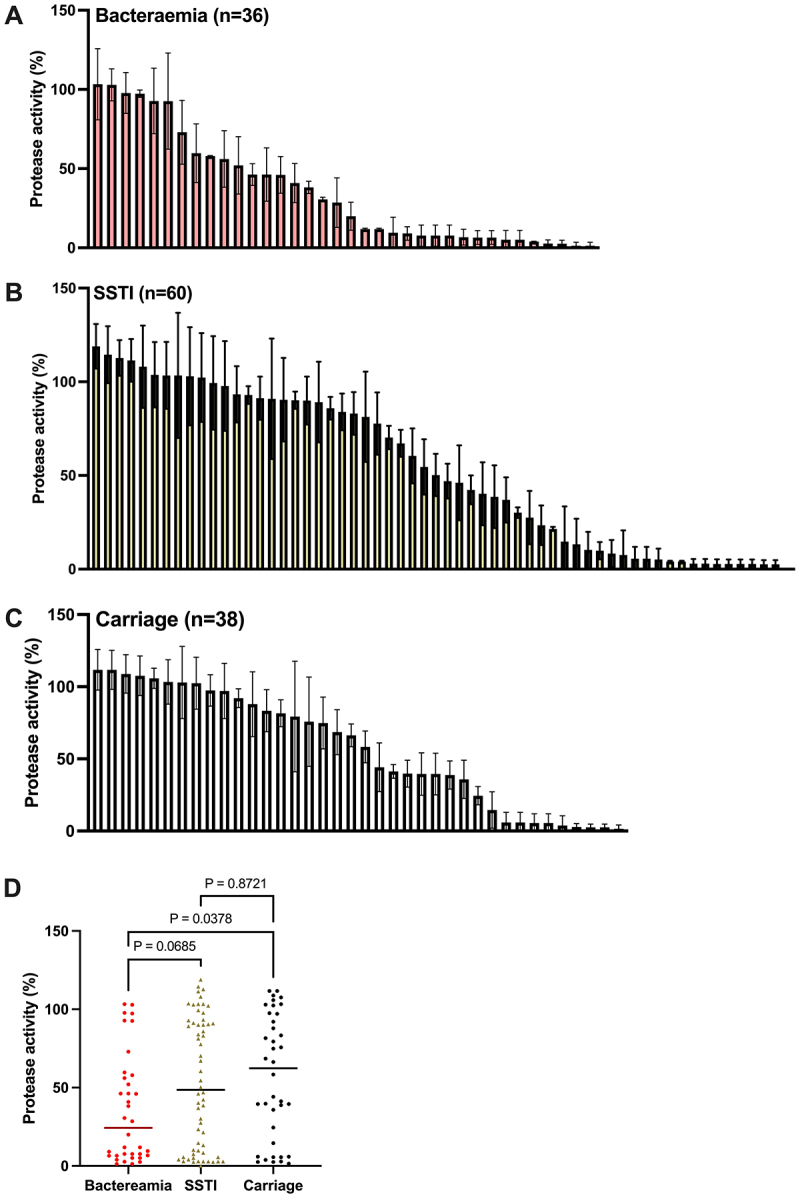
Casein hydrolysis assay comparing protease activity of 134 *S. aureus* USA300 clinical isolates. A) bacteraemia, B) skin and soft tissue infection (SSTI) and C) carriage clinical isolates were assessed for protease production. Diameter of hydrolysis was calculated, and percentage protease activity was determined using strain LAC as 100% and the complete protease null strain as 0%. Three biological repeats were included. These figures visually display the mean and standard deviation from three repeats. D) the protease activity of 134 clinical isolates. Each dot represents average protease activity of each isolate. Statistical differences were calculated by non-parametric one-way ANOVA.

The protease activity of clinical isolates varied significantly across the different infection sources, ranging from complete absence of protease secretion to > 115% protease activity between the lowest and highest isolates (Figure 2A-C). Forty-nine isolates exhibited poor protease production, falling below 20% protease activity, whereas 6 isolates showed high protease expression, surpassing 110% protease activity. To determine the correlation between protease activity and infection severity, we compared relative protease activity from the three different clinical sources. Bacteraemia isolates had the lowest combined protease activity, significantly lower than the carriage group (*p* = 0.0379), but not significantly different to the SSTI cohort (*p* = 0.0685). This indicated a negative correlation between protease activity and invasive disease (Figure 2D).

To determine whether low protease activity resulted in enhanced biofilm formation which may be linked to increased bloodstream infection, we tested the biofilm forming capacity of 15 high ( > 90% protease activity) and 15 low protease ( <5% protease activity) strains for biofilm formation as previously described [29] using the crystal violet method. No significant difference between the low and high protease forming group was identified (Supplementary Figure 2A).

Inactivation of the ClpXP protease, an AAA+ protease, that comprises ATP-dependent unfoldase and polypeptide translocase ClpX, and self-compartmentalized peptidase ClpP [30], has been observed to increase β-lactam resistance in USA300 [31]. To determine whether differences in the level of secreted proteases impacted β-lactam susceptibility, we compared the MIC of all bloodstream infections against the β-lactam oxacillin. Spearman test showed no correlation between oxacillin MIC and protease activity (*r* = 0.058; *p* = 0.73); with the vast majority of isolates displayed an MIC of 16–32 µg/ml (Supplementary Figure 2B).

To determine whether extracellular protease activity correlated with other virulence factors, we conducted a comparative analysis of protease activity and toxicity, a virulence factor trait that was previously measured using the same collection of isolates [20]. Spearman analysis revealed no correlation between protease activity and toxicity (r coefficient = 0.05830 (−0.1171 to 0.2307)).

**Figure 3.**
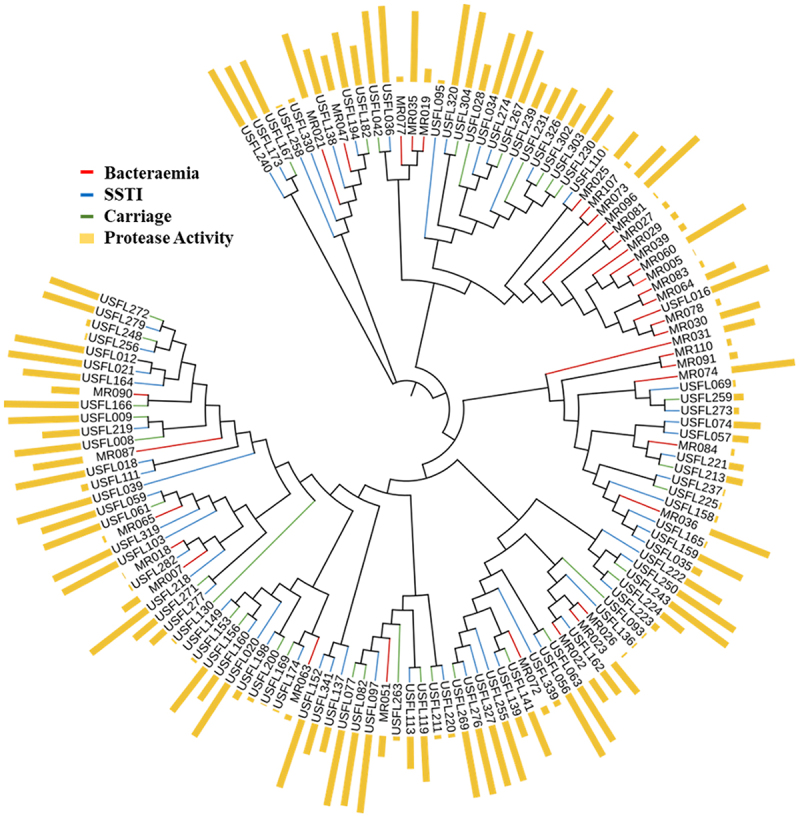
Protease expression and infection source mapped across the cohort of USA300 isolates. A SNP-based maximum likelihood tree of 134 isolates revealed the broad distribution of protease activity. The data presented as column is the percentage of protease activity for each isolate. Branch colours describe different disease origins inferred; red = bacteraemia; blue = SSTI; green = carriage. External bars represent the normalised protease activity.

To better understand how differences in protease activity are distributed across the genetic variability that exists within this collection of clinical isolates, we analysed genome sequences using a maximum likelihood tree based on the alignments of core *S. aureus* SNPs (Figure 3). A broad distribution of protease phenotypes was observed across the collection with multiple examples of highly related isolates displaying similar and very different protease expression profiles.

### Mutations in a broad range of loci alter protease expression in S. aureus

**Figure 4.**
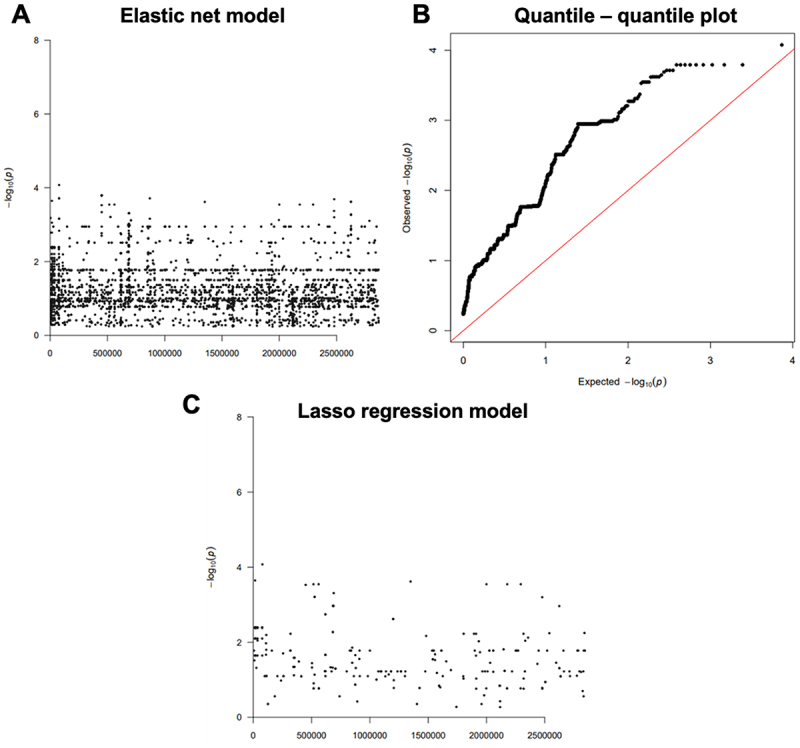
Genes associated with protease activity in genome-wide association studies. A) the Manhattan plot for the association of *k*-mers with protease activity using an elastic net regression model. B) the quantile-quantile plot of the observed *p*-values indicating lineage effect. C) the Manhattan plot for the association of *k*-mers with protease activity using a lasso regression model.

To identify the genetic polymorphisms responsible for the broad variation observed in protease expression, we adopted a functional genomics strategy, employing GWAS to identify polymorphisms associated with proteolytic activity. GWAS is commonly performed using SNP-based systems. However, these strategies ignore structural variations such as gene insertions, deletions, and copy number variations and require a completed reference genome. These factors may introduce biases in variant calling [32]. In this study, we performed alignment free methods, which identified variable length DNA sequences (*k*-mers) associated with protease activity with mapping using the *S. aureus* pangenome. The elastic net model was used to test the association between *k*-mer and protease activity. This model employs regularized linear regression to simultaneously assess the association of all genetic variants with the phenotype, providing a report of the predicted causal variants for the phenotype (Figure 4A). The quantile-quantile plot of the observed *p*-value in elastic net model shows the presence of lineage effects, suggesting that variation within multiple loci is associated with the phenotype, without all variation necessarily being causal for the phenotype (Figure 4B). Thus, the causal variants were further condensed to a single locus that has the largest effect on protease activity using a lasso regression model (Figure 4C).

Numerous unique *k*-mers were found to be significantly associated with protease activity. Using basic local alignment search tool (BLAST), significant *k*-mers were mapped to 68 genes present in the JE2/LAC genome, the wild-type reference strain of the NTML and lineage used in the screening of clinical isolates (Supplementary Table 2). Of the 68 genes identified, 51 has corresponding transposon mutants available from the NTML library. One gene that significantly associated with protease activity following our GWAS analysis was *agrA*, that we (Figure 1B) and others [18,33,34] have previously shown to have a functional association with protease activity, providing some proof of principle for the accuracy of our approach.

### Functional verification of genes associated with protease expression identified by GWAS

**Figure 5.**
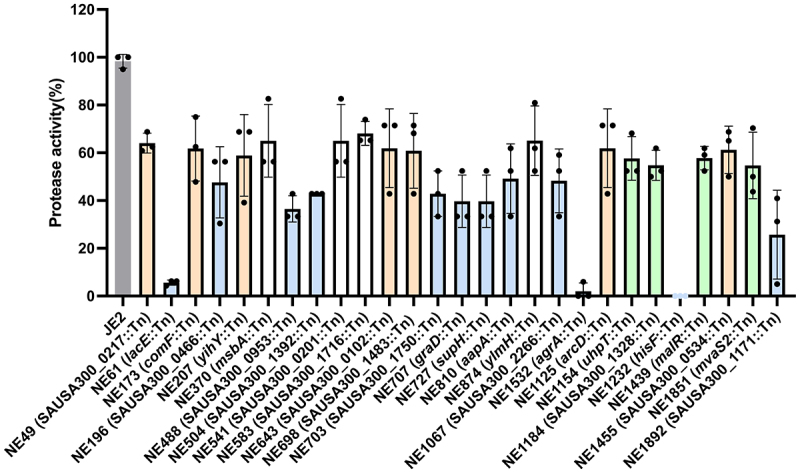
Functional validation of protease activity associated genes. Transposon insertion mutants were assessed for protease activity using the casein hydrolysis assay and compared to the wildtype JE2 and normalised against strain LAC and the complete protease null strain AH1919. Three biological repeats were included. Each repeat is represented by dots and errors bars represent the standard deviation. Statistical differences were calculated by one-way ANOVA. Blank-filled bars represent *p< 0.05, orange-filled bars represent **p< 0.01, green-filled bars represent ***p< 0.001, blue-filled bars represent ****p<0.0001.

False positive association between genetic factors and phenotypes is a common issue with GWAS. These arise due to several factors, including correlation between casual factors and noncausal markers, presence of more than a single causal factor and epistasis [35]. Typically, error is the result of two related things: lineage effects and the low power because of limited replicates. The latter is exacerbated when limiting analyses to SNPs as genes may or may not be present, k-mer methods were designed to account for this in bacterial genomes. A key feature of contemporary bacterial GWAS studies is laboratory validation of in silico findings [36–38]. Thus, in order to validate the importance of GWAS-identified *k*-mers on protease activity and to estimate the rate of false positive associations generated in our analysis, we used the NTML to verify the function of genes containing associated *k-*mers in relation to protease activity. We analysed the impact on transposon-mediated interruption of gene function representing those genes in which there was an available transposon mutant in the NTML. Of the 51 transposon mutants examined, 27 were associated with a statistically significant reduction in protease activity (Figure 5; Table 1). The genes associated with reduction were annotated as conserved hypothetical proteins, hydrolases, membrane proteins, drug transporters, permeases, and transcription factors. In addition to *agrA*, our GWAS identified *k*-mers mapped to SAUSA300_0217 which codes for an AraC family DNA-binding response regulator, which was also previously reported to have decrease in protease activity when disrupted via transposon insertion [18]. Apart from *agrA* and SAUSA300_0217, the other 25 genes, as far as we are aware, have not been described in the literature to be associated with protease expression.

**Table 1.** Functional validated genes associated with protease activity.

NTML	Gene	Gene description	Impact	*p* value
NE49	SAUSA300_0217	DNA-binding response regulator, AraC family	Decreased	0.0123
NE61	*lacE*	PTS system, lactose-specific IIBC component	Decreased	<0.0001
NE173	*comF*	Putative comf operon protein 1	Decreased	0.0051
NE196	SAUSA300_0466	Conserved hypothetical protein	Decreased	<0.0001
NE207	*yihY*	Putative membrane protein	Decreased	0.0016
NE370	*msbA*	Conserved hypothetical protein	Decreased	0.0179
NE488	SAUSA300_0953	Putative membrane protein	Decreased	<0.0001
NE504	SAUSA300_1392	PhiSLT ORF191-like protein	Decreased	<0.0001
NE541	SAUSA300_0201	Peptide ABC transporter, permease protein	Decreased	0.0179
NE583	SAUSA300_1716	Conserved hypothetical protein	Decreased	0.0303
NE643	SAUSA300_0102	Staphylococcal tandem lipoprotein	Decreased	0.0054
NE698	SAUSA300_1483	Conserved hypothetical protein	Decreased	0.0035
NE703	SAUSA300_1750	Conserved hypothetical protein	Decreased	<0.0001
NE707	*graD*	Putative lipoprotein	Decreased	<0.0001
NE727	*supH*	Hydrolase, haloacid dehalogenase-like family	Decreased	<0.0001
NE810	*aapA*	D-serine/D-alanine/glycine transporter	Decreased	<0.0001
NE874	*ylmH*	Hypothetical protein	Decreased	0.0182
NE1067	SAUSA300_2266	Hypothetical protein	Decreased	<0.0001
NE1125	*arcD*	Arginine/oirnithine antiporter	Decreased	0.0054
NE1154	*uhpT*	Sugar phosphate antiporter	Decreased	0.0009
NE1184	SAUSA300_1328	Putative drug transporter	Decreased	0.0003
NE1232	*hisF*	Imidazole glycerol phosphate synthase subunit HisF	Decreased	<0.0001
NE1439	*malR*	Maltose operon transcriptional repressor	Decreased	0.0010
NE1455	SAUSA300_0534	Amidohydrolase	Decreased	0.0041
NE1532	*agrA*	Accessory gene regulator protein A	Decreased	<0.0001
NE1851	*mvaS2*	Hypothetical protein	Decreased	0.0002
NE1892	SAUSA300_1171	Hypothetical protein	Decreased	<0.0001

NTML refers to the transposon mutant available in the Nebraska Transposon mutant library [19]. Impact refers to the alteration in protease activity resulting from the disruption of the indicated gene. *p* value calculated by one-way ANOVA vs wildtype JE2 strain.

## Discussion

The extracellular proteases of *S. aureus* play a crucial role in pathogenesis, possessing the combined capacity to hydrolyse host proteins for nutrition and immune evasion purposes while simultaneously re-modelling virulence factor stability and abundance through targeted degradation. We have shown that the casein hydrolysis assay reports on the activity of four main staphylococcal proteases (Aur, SspB, SspA, and SplF) but is limited, providing gross proteolytic activity rather than reporting on individual proteases. We did not observe any decrease in proteolytic activity when using *scpA*::Tn and *splB-E*::Tn mutants, however we have not ruled out that under our *in vitro* culture conditions these proteases may not be secreted in high abundance and therefore deletion of these genes results in no measurable or significant difference compared to the WT strain. In addition, we observed a disagreement with respect to SarS and Rot mediated protease regulation, whereby inactivation of *sarS* or *rot* resulted in no measurable difference in protease activity (Figure 1B). This is in contrast to certain published studies; Oscarsson et al. showed that the overexpression of *rot* in highly RNAIII (Agr++) active strains or inactivation of *rot* in strains with low levels of RNAIII (Agr-) impacted *aur* and *sspA* expression [39]. Our study differs from that of the Oscarsson study in that we have focused on USA300 strain JE2 which is a strong Agr/RNAIII expression strain, and therefore deletion of *rot* may not have a significant impact on *aur* or *sspA* expression and therefore would not be evident using a phenotypic screen of protease activity. However, Mootz et al. identified *rot* as a repressor of protease expression in a USA300 strain and activity levels of the cysteine proteases were increased in a *rot* mutant which resulted in a lower biofilm capacity in the mutant strain [40]. In contrast we and Fey et al [19] reported that a *rot*::Tn or *SarS*::Tn mutant resulted in no alteration in protease activity using the casein hydrolysis assay readout and the same *S. aureus* strain background (JE2).

It is important to note that deletion of virulence regulators such as Rot, SarS, and others has been shown to result in the up and down regulation of individual proteases [18]. Thus, employing assays to measure cumulative protease activity between WT and isogenic mutants, as examined in this study, may remain unchanged due to differential expression of individual proteases arising following mutation/inactivation which offer no significant difference in combined protease activity when measured phenotypically. Furthermore, strain-specificity, culture conditions and differences in genotypic and phenotypic proteases read-out may also contribute to differences observed when specific virulence genes are interrogated for their role in regulating specific proteases in *S. aureus*.

The greatest decrease in proteolytic activity was observed with the *aur*::Tn mutant. Aur is secreted as a zymogen which undergoes autocatalytic cleavage to become active [41]. Aur has low substrate specificity which facilitates its role in both the maturation and degradation of numerous *S. aureus* proteins. SspA and SspB are also initially produced as zymogens and their activation relies on the processing activity of Aur. Aur cleaves SspA, leading to the subsequent activation of SspB by SspA [5]. Therefore, the *aur*::Tn mutant is likely to be defective in active Aur, SspB and SspA.

Applying GWAS to identify genes associated with protease expression in *S. aureus* identified previously unknown phenotype-linked variation. We chose to concentrate on a cohort of genetically related clinical isolates belonging to the USA300 North American epidemic clone, which has remained a dominant healthcare and community associated MRSA in both North and South America [42]. We analysed 134 isolates from three different clinical origins and observed significant variation in protease activity and report a statistically significant decrease in protease expression in bacteraemia isolates compared to carriage isolates. Using the same isolate collection, we have previously shown that toxin expression from blood strains was negatively correlated with disease severity. In addition, we observed that lower toxic isolates were relatively more fit when grown in human serum compared to high toxin isolates, providing an explanation for the unexpected association of low toxic isolates with invasive disease [20]. We investigated whether there was any correlation between individual isolate protease and toxin phenotypes. Surprisingly we found no significant correlation, indicating that different genetic variants are at play that result in the variation in proteolysis and toxicity. Mapping the protease activity onto a maximum likelihood tree constructed from the genome sequences of these related isolates revealed a wide variation of protease activity across genotypes with some clustering evident. Importantly, there were several examples of highly related strains that differed significantly in protease activity, most notably strain pairs MR035/MR019; MR073/MR107; USFL159/USFL035; USFL141/USFL139; MR090/USFL166 and USFL018/USFL111.

Our GWAS results identified *k*-mers mapped to 68 genes that were significantly related to alteration in protease activity. Using the NTML library as a tool to understand the importance of gene disruption on identified genes, we observed that 27 from the available 51 transposon mutants of our GWAS candidate associated genes had reduced protease activity, giving a positive hit rate of 53% (27/51). This is significantly better than our previous GWAS analyses that used SNP-based systems [20,21,43]. These validated loci were annotated as conserved hypothetical proteins, hydrolases, membrane proteins, drug transporters, permeases, and an AraC family DNA-binding response regulator that has been previously observed to be associated with protease expression in *S. aureus* [18]. Seventeen genes identified as associated with protease activity had no available Tn mutant. Ten genes (identified in bold in (Supplementary Table 2)) were reported to be essential [19,44] which included SAUSA300_0005 (DNA gyrase subunit B, *gyrB*), SAUSA300_0760 (phosphopyruvate hydratase, *eno*), SAUSA300_0818 (FeS assembly ATPase, *sufC*) SAUSA300_0822 (FeS assembly protein SufB, *sufB*), SAUSA300_1022 (hypothetical protein), SAUSA300_1143(DNA topoisomerase I, *topA*) SAUSA300_1446 (hypothetical protein), SAUSA300_1893 (NAD synthetase, *nadE*), SAUSA300_2104 (glucosamine-fructose-6-phosphate aminotransferase, *glmS*), SAUSA300_2141 (IS5/IS1182 family transposase). The remaining seven genes were not included in the NTML, most likely due to the transposon mutant that was generated did not meet the criteria for inclusion in the library [44].

As well as previously characterized genes linked to protease expression, the GWAS analyses identified several novel loci, indicating that the regulation of extracellular proteases in *S. aureus* clinical isolates is highly complex. The emergence of metabolism-related loci, including *lacE*, associated with lactose metabolism, and *malR*, related to maltose metabolism, in our GWAS results strengthens the link that alterations in central metabolism significantly impact virulence [45]. Additionally, *uhpT*, a glucose-6-phosphate transporter and part of the hexose phosphate transport (Hpt) system, which has been previously associated with fosfomycin resistance [46], was identified in this study to play a role in extracellular protease activity. Moreover, *arcD*, an arginine/ornithine antiporter and member of the arginine deiminase (arc) operon which has been reported to play a role in the production and accumulation of the polysaccharide intracellular adhesin [47] also caused decreased protease secretion when disrupted. Lastly, another highly significant GWAS hit involved *hisF*, an imidazole glycerol phosphate synthase involved in histidine biosynthesis and connected to nitrogen metabolism. Information on the role of HisF in *S. aureus* pathophysiology is lacking. However, HisF has been reported to be essential for full virulence in an *Acinetobacter baumannii* murine pneumonia model where disruption of *hisF* resulted in enhanced survival of mice (Martínez-Guitián et al., 2019). The role the other identified genes (Table 1) plays in protease expression is currently unclear but work to unravel their contribution is ongoing.

The identification of new loci involved in protease regulation expands understanding of the complex regulation of protease expression and adds to the increasing knowledge on the relationship between metabolism and virulence in *S. aureus*. These new insights have the potential to contribute to the development of novel anti-virulence therapies targeting specific pathways involved in the production of proteases, a critical feature of *S. aureus* pathogenesis.

## Supplementary Material

Supplementary Material_Li et al 2025.docx

## Data Availability

The data that support the findings of this study are openly available at figshare at http://doi.org/10.6084/m9.figshare.28203137.
