# Supplementary material for: Genome-wide association analysis provides insights into the genomics and extracellular expression of Staphylococcus aureus proteases
Source: Virulence. 2025 Aug 3;16(1):2543116. doi: 10.1080/21505594.2025.2543116 (PMC12355673; doi:10.1080/21505594.2025.2543116)
Supplement: Supplementary Material_Li et al 2025.docx [file KVIR_A_2543116_SM9781.docx]

**Supplementary Material**

**Genome-wide association analysis provides insights into the genomics and extracellular expression of *Staphylococcus aureus* proteases**

Shuxian Li^a^, William Monteith^b^, Emily Rudolph^a^, Samuel K Sheppard^b^, Maisem Laabei^a,c*^

^a^ Department of Life Sciences, University of Bath, Bath, BA2 7AY, UK.

^b^ Ineos Oxford Institute, Department of Biology, University of Oxford, Oxford, OX1 3SZ, UK

^c^ School of Cellular and Molecular Medicine, University of Bristol, Bristol, BS8 1TD, UK.

* Corresponding author: [Maisem.laabei@bristol.ac.uk](mailto:Maisem.laabei@bristol.ac.uk)

**Supplementary Figure 1:**

**A)**

**
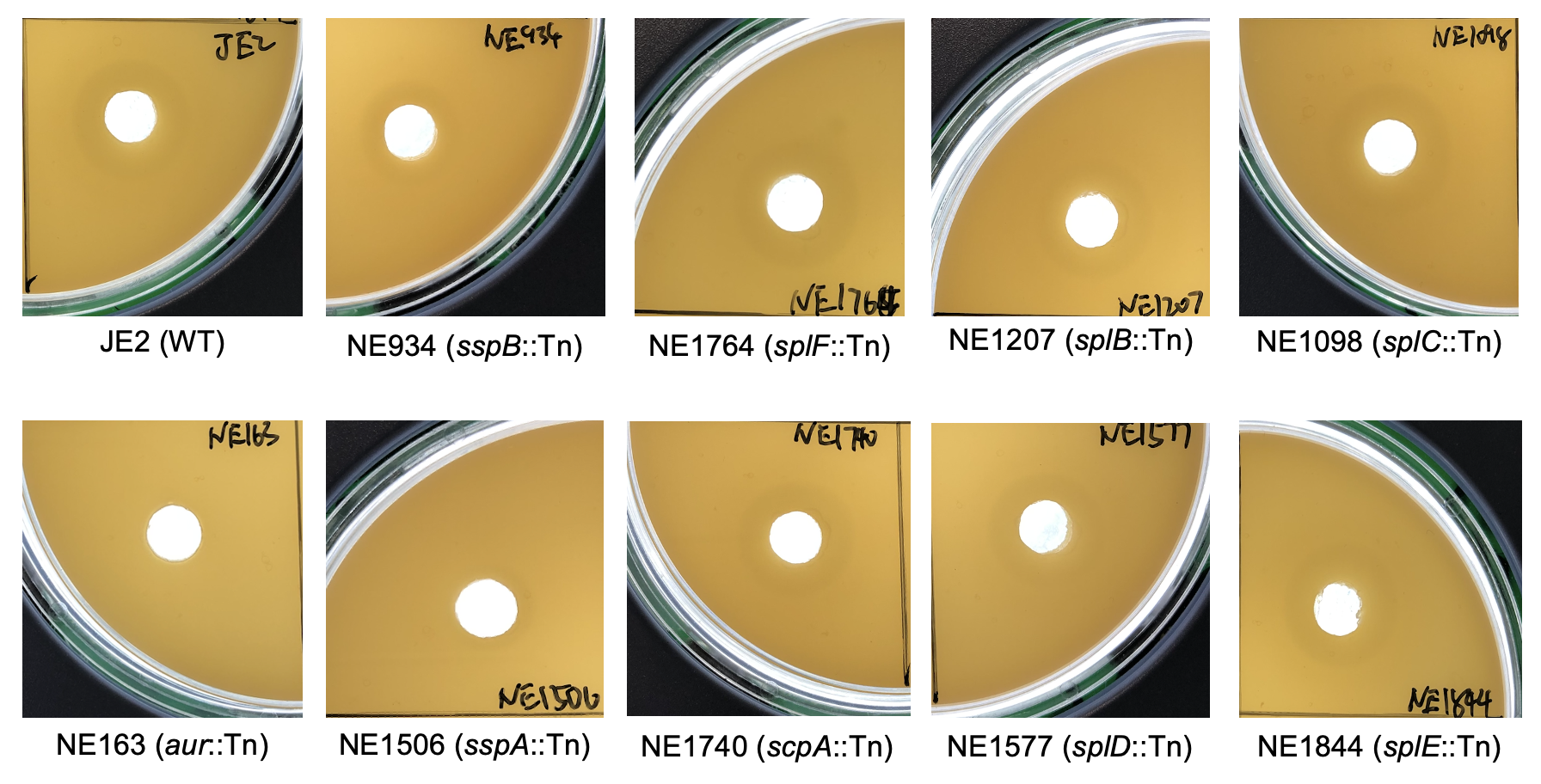
**

**B)**

**
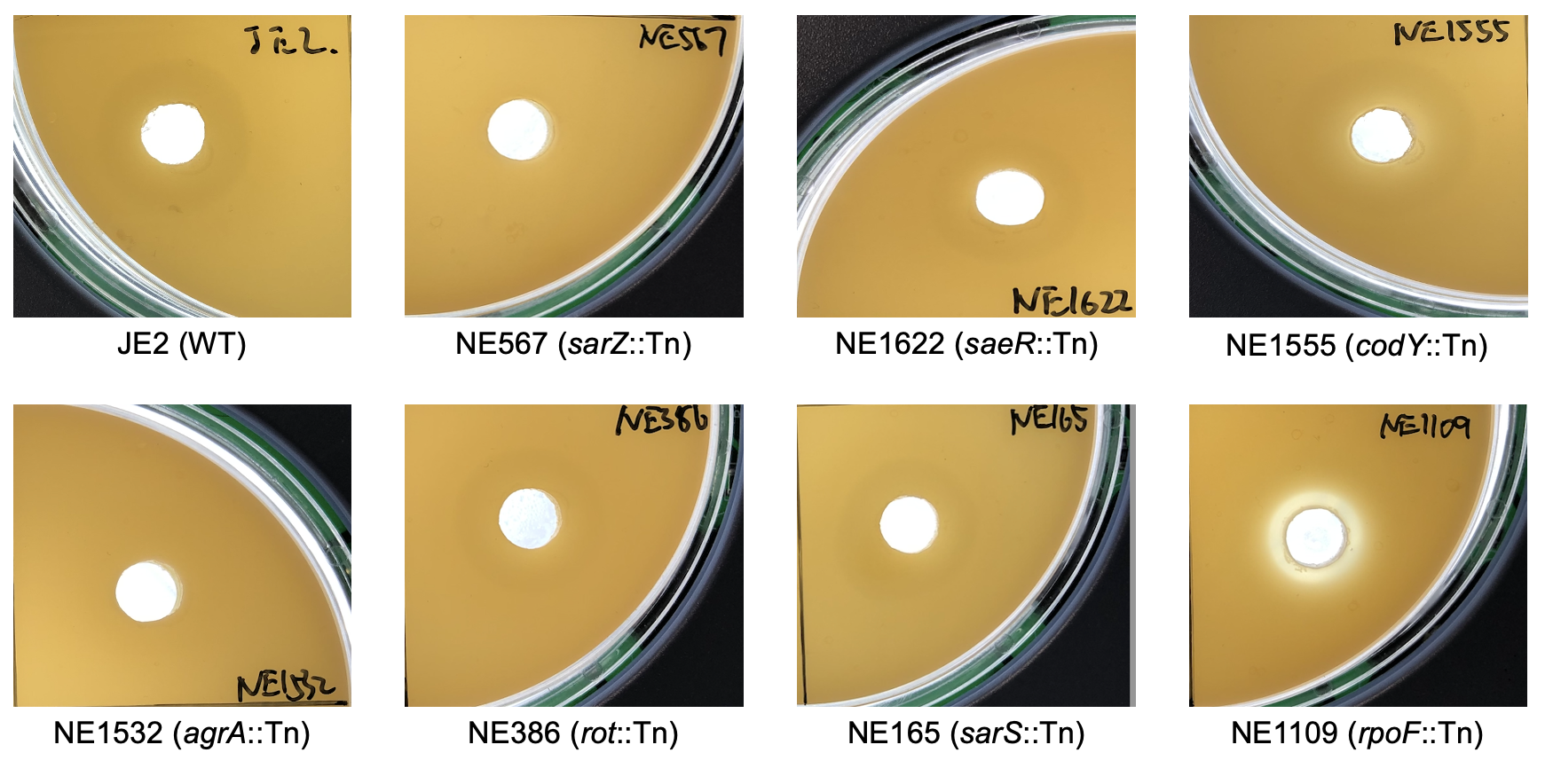
**

**C)
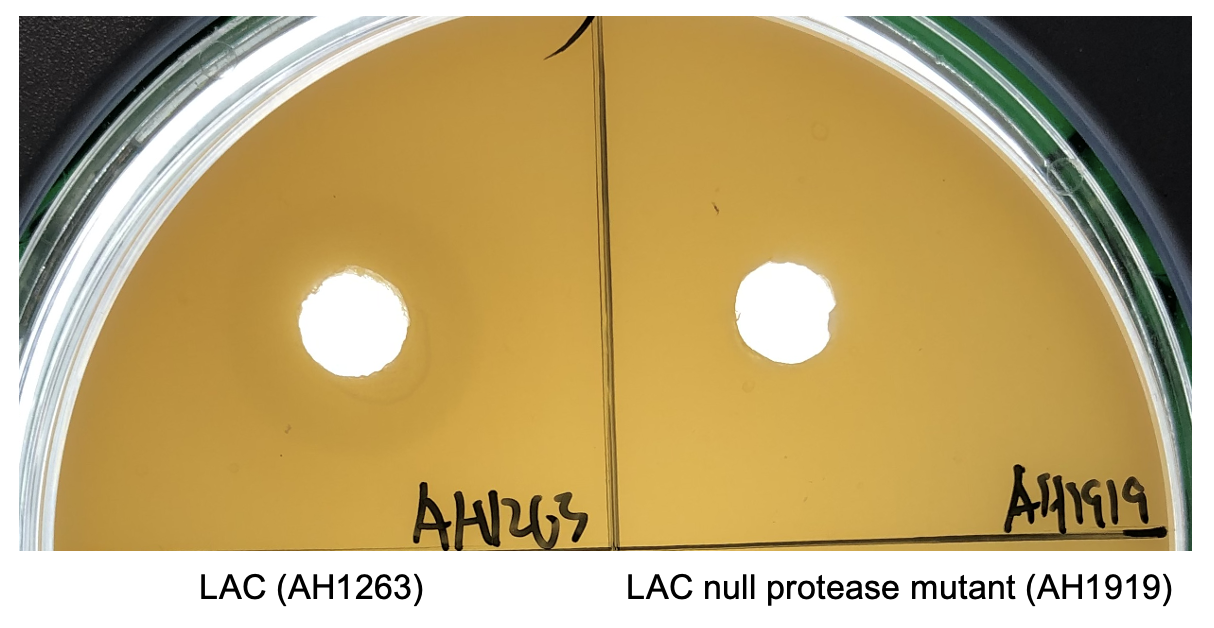
**

**Supplementary Figure 1: Images of casein hydrolysis assay.**

Transposon insertion mutants of **A)** protease mutants and **B)** virulence gene regulators. Diameter of hydrolysis was calculated, and percentage protease activity was determined using **C)** strain AH1263 as 100% and strain AH1919 as 0%.

**Supplementary Figure 2:**

**
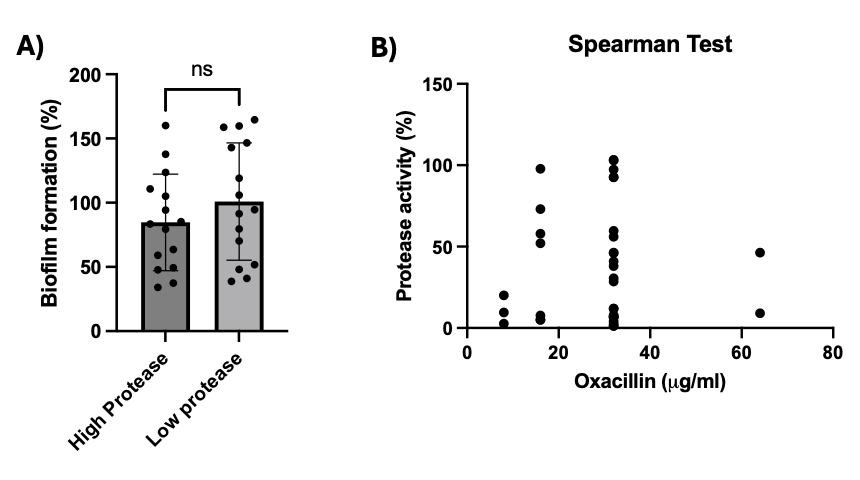
**

**Supplementary Figure 2: Association of protease activity with biofilm formation and oxacillin sensitivity**

**A)** Biofilm forming capacity after 24h growth of 15 high and 15 low protease CC8 strains were examined using the crystal violet method and compared to biofilm positive and negative control strains as described (1). **B)** Oxacillin susceptibility using standard MIC conditions (2)was performed on all bloodstream infection isolates and correlation between protease expression and antibiotic susceptibility was performed using spearman correlation test.

**Supplementary Table 1: List of strains used in this study.**

| **Isolate / reference** | **Genotype** | **Infectious source** | **Reference or ENA accession no.** |
| --- | --- | --- | --- |
| MR005 (3) | CC8 USA300 | Blood (bacteraemia) | ERR449858 |
| MR007 (3) | CC8 USA300 | Blood (bacteraemia) | ERR449859 |
| MR018 (3) | CC8 USA300 | Blood (bacteraemia) | ERR449860 |
| MR019 (3) | CC8 USA300 | Blood (bacteraemia) | ERR449861 |
| MR021 (3) | CC8 USA300 | Blood (bacteraemia) | ERR449862 |
| MR022 (3) | CC8 USA300 | Blood (bacteraemia) | ERR449863 |
| MR023 (3) | CC8 USA300 | Blood (bacteraemia) | ERR449864 |
| MR025 (3) | CC8 USA300 | Blood (bacteraemia) | ERR449865 |
| MR026 (3) | CC8 USA300 | Blood (bacteraemia) | ERR449866 |
| MR027 (3) | CC8 USA300 | Blood (bacteraemia) | ERR449867 |
| MR029 (3) | CC8 USA300 | Blood (bacteraemia) | ERR449868 |
| MR030 (3) | CC8 USA300 | Blood (bacteraemia) | ERR449869 |
| MR031 (3) | CC8 USA300 | Blood (bacteraemia) | ERR449870 |
| MR035 (3) | CC8 USA300 | Blood (bacteraemia) | ERR449871 |
| MR036 (3) | CC8 USA300 | Blood (bacteraemia) | ERR449872 |
| MR039 (3) | CC8 USA300 | Blood (bacteraemia) | ERR449873 |
| MR047 (3) | CC8 USA300 | Blood (bacteraemia) | ERR449874 |
| MR051 (3) | CC8 USA300 | Blood (bacteraemia) | ERR449875 |
| MR060 (3) | CC8 USA300 | Blood (bacteraemia) | ERR449876 |
| MR063 (3) | CC8 USA300 | Blood (bacteraemia) | ERR449877 |
| MR064 (3) | CC8 USA300 | Blood (bacteraemia) | ERR449878 |
| MR065 (3) | CC8 USA300 | Blood (bacteraemia) | ERR449879 |
| MR072 (3) | CC8 USA300 | Blood (bacteraemia) | ERR449880 |
| MR073 (3) | CC8 USA300 | Blood (bacteraemia) | ERR449881 |
| MR074 (3) | CC8 USA300 | Blood (bacteraemia) | ERR449882 |
| MR077 (3) | CC8 USA300 | Blood (bacteraemia) | ERR449883 |
| MR078 (3) | CC8 USA300 | Blood (bacteraemia) | ERR449884 |
| MR081 (3) | CC8 USA300 | Blood (bacteraemia) | ERR449885 |
| MR083 (3) | CC8 USA300 | Blood (bacteraemia) | ERR449886 |
| MR084 (3) | CC8 USA300 | Blood (bacteraemia) | ERR449887 |
| MR087 (3) | CC8 USA300 | Blood (bacteraemia) | ERR449888 |
| MR090 (3) | CC8 USA300 | Blood (bacteraemia) | ERR449889 |
| MR091 (3) | CC8 USA300 | Blood (bacteraemia) | ERR449890 |
| MR096 (3) | CC8 USA300 | Blood (bacteraemia) | ERR449891 |
| MR107 (3) | CC8 USA300 | Blood (bacteraemia) | ERR449892 |
| MR110 (3) | CC8 USA300 | Blood (bacteraemia) | ERR449893 |
| USFL008 (4) | CC8 USA300 | Nose/skin (carriage) | ERS092765 |
| USFL009 (4) | CC8 USA300 | Nose/skin (carriage) | ERS092766 |
| USFL012 (4) | CC8 USA300 | Nose/skin (carriage) | ERS092769 |
| USFL028 (4) | CC8 USA300 | Nose/skin (carriage) | ERS092786 |
| USFL042 (4) | CC8 USA300 | Nose/skin (carriage) | ERS092800 |
| USFL061 (4) | CC8 USA300 | Nose/skin (carriage) | ERS092819 |
| USFL063 (4) | CC8 USA300 | Nose/skin (carriage) | ERS092821 |
| USFL074 (4) | CC8 USA300 | Nose/skin (carriage) | ERS092833 |
| USFL077 (4) | CC8 USA300 | Nose/skin (carriage) | ERS092836 |
| USFL082 (4) | CC8 USA300 | Nose/skin (carriage) | ERS092841 |
| USFL093 (4) | CC8 USA300 | Nose/skin (carriage) | ERS092852 |
| USFL119 (4) | CC8 USA300 | Nose/skin (carriage) | ERS092879 |
| USFL130 (4) | CC8 USA300 | Nose/skin (carriage) | ERS092890 |
| USFL141 (4) | CC8 USA300 | Nose/skin (carriage) | ERS092901 |
| USFL153 (4) | CC8 USA300 | Nose/skin (carriage) | ERS092913 |
| USFL156 (4) | CC8 USA300 | Nose/skin (carriage) | ERS092916 |
| USFL166 (4) | CC8 USA300 | Nose/skin (carriage) | ERS092927 |
| USFL167 (4) | CC8 USA300 | Nose/skin (carriage) | ERS092928 |
| USFL169 (4) | CC8 USA300 | Nose/skin (carriage) | ERS092930 |
| USFL182 (4) | CC8 USA300 | Nose/skin (carriage) | ERS092943 |
| USFL200 (4) | CC8 USA300 | Nose/skin (carriage) | ERS092961 |
| USFL211 (4) | CC8 USA300 | Nose/skin (carriage) | ERS092973 |
| USFL213 (4) | CC8 USA300 | Nose/skin (carriage) | ERS092975 |
| USFL224 (4) | CC8 USA300 | Nose/skin (carriage) | ERS092986 |
| USFL225 (4) | CC8 USA300 | Nose/skin (carriage) | ERS092987 |
| USFL230 (4) | CC8 USA300 | Nose/skin (carriage) | ERS092992 |
| USFL231 (4) | CC8 USA300 | Nose/skin (carriage) | ERS092993 |
| USFL243 (4) | CC8 USA300 | Nose/skin (carriage) | ERS093005 |
| USFL248 (4) | CC8 USA300 | Nose/skin (carriage) | ERS093010 |
| USFL259 (4) | CC8 USA300 | Nose/skin (carriage) | ERS093022 |
| USFL263 (4) | CC8 USA300 | Nose/skin (carriage) | ERS093026 |
| USFL267 (4) | CC8 USA300 | Nose/skin (carriage) | ERS093030 |
| USFL269 (4) | CC8 USA300 | Nose/skin (carriage) | ERS093032 |
| USFL271 (4) | CC8 USA300 | Nose/skin (carriage) | ERS093034 |
| USFL272 (4) | CC8 USA300 | Nose/skin (carriage) | ERS093035 |
| USFL302 (4) | CC8 USA300 | Nose/skin (carriage) | ERS093066 |
| USFL303 (4) | CC8 USA300 | Nose/skin (carriage) | ERS093067 |
| USFL304 (4) | CC8 USA300 | Nose/skin (carriage) | ERS093068 |
| USFL016 (4) | CC8 USA300 | Abscess (SSTI) | ERS092771 |
| USFL018 (4) | CC8 USA300 | Abscess (SSTI) | ERS092775 |
| USFL020 (4) | CC8 USA300 | Abscess (SSTI) | ERS092778 |
| USFL021 (4) | CC8 USA300 | Abscess (SSTI) | ERS092779 |
| USFL034 (4) | CC8 USA300 | Abscess (SSTI) | ERS092792 |
| USFL035 (4) | CC8 USA300 | Abscess (SSTI) | ERS092793 |
| USFL036 (4) | CC8 USA300 | Abscess (SSTI) | ERS092794 |
| USFL039 (4) | CC8 USA300 | Abscess (SSTI) | ERS092797 |
| USFL056 (4) | CC8 USA300 | Abscess (SSTI) | ERS092814 |
| USFL057 (4) | CC8 USA300 | Abscess (SSTI) | ERS092815 |
| USFL059 (4) | CC8 USA300 | Abscess (SSTI) | ERS092817 |
| USFL069 (4) | CC8 USA300 | Abscess (SSTI) | ERS092828 |
| USFL095 (4) | CC8 USA300 | Abscess (SSTI) | ERS092854 |
| USFL097 (4) | CC8 USA300 | Abscess (SSTI) | ERS092856 |
| USFL103 (4) | CC8 USA300 | Abscess (SSTI) | ERS092862 |
| USFL110 (4) | CC8 USA300 | Abscess (SSTI) | ERS092867 |
| USFL111 (4) | CC8 USA300 | Abscess (SSTI) | ERS092870 |
| USFL113 (4) | CC8 USA300 | Abscess (SSTI) | ERS092873 |
| USFL136 (4) | CC8 USA300 | Abscess (SSTI) | ERS092896 |
| USFL137 (4) | CC8 USA300 | Abscess (SSTI) | ERS092897 |
| USFL138 (4) | CC8 USA300 | Abscess (SSTI) | ERS092898 |
| USFL139 (4) | CC8 USA300 | Abscess (SSTI) | ERS092899 |
| USFL149 (4) | CC8 USA300 | Abscess (SSTI) | ERS092909 |
| USFL152 (4) | CC8 USA300 | Abscess (SSTI) | ERS092912 |
| USFL158 (4) | CC8 USA300 | Abscess (SSTI) | ERS092918 |
| USFL159 (4) | CC8 USA300 | Abscess (SSTI) | ERS092919 |
| USFL160 (4) | CC8 USA300 | Abscess (SSTI) | ERS092920 |
| USFL162 (4) | CC8 USA300 | Abscess (SSTI) | ERS092922 |
| USFL164 (4) | CC8 USA300 | Abscess (SSTI) | ERS092925 |
| USFL165 (4) | CC8 USA300 | Abscess (SSTI) | ERS092926 |
| USFL173 (4) | CC8 USA300 | Abscess (SSTI) | ERS092934 |
| USFL174 (4) | CC8 USA300 | Abscess (SSTI) | ERS092935 |
| USFL194 (4) | CC8 USA300 | Abscess (SSTI) | ERS092955 |
| USFL198 (4) | CC8 USA300 | Abscess (SSTI) | ERS092959 |
| USFL218 (4) | CC8 USA300 | Abscess (SSTI) | ERS092980 |
| USFL219 (4) | CC8 USA300 | Abscess (SSTI) | ERS092981 |
| USFL220 (4) | CC8 USA300 | Abscess (SSTI) | ERS092982 |
| USFL221 (4) | CC8 USA300 | Abscess (SSTI) | ERS092983 |
| USFL222 (4) | CC8 USA300 | Abscess (SSTI) | ERS092984 |
| USFL223 (4) | CC8 USA300 | Abscess (SSTI) | ERS092985 |
| USFL237 (4) | CC8 USA300 | Abscess (SSTI) | ERS092999 |
| USFL239 (4) | CC8 USA300 | Abscess (SSTI) | ERS093001 |
| USFL240 (4) | CC8 USA300 | Abscess (SSTI) | ERS093002 |
| USFL250 (4) | CC8 USA300 | Abscess (SSTI) | ERS093012 |
| USFL255 (4) | CC8 USA300 | Abscess (SSTI) | ERS093017 |
| USFL256 (4) | CC8 USA300 | Abscess (SSTI) | ERS093018 |
| USFL258 (4) | CC8 USA300 | Abscess (SSTI) | ERS093021 |
| USFL273 (4) | CC8 USA300 | Abscess (SSTI) | ERS093036 |
| USFL274 (4) | CC8 USA300 | Abscess (SSTI) | ERS093037 |
| USFL276 (4) | CC8 USA300 | Abscess (SSTI) | ERS093039 |
| USFL277 (4) | CC8 USA300 | Abscess (SSTI) | ERS093040 |
| USFL279 (4) | CC8 USA300 | Abscess (SSTI) | ERS093042 |
| USFL282 (4) | CC8 USA300 | Abscess (SSTI) | ERS093045 |
| USFL319 (4) | CC8 USA300 | Abscess (SSTI) | ERS093083 |
| USFL320 (4) | CC8 USA300 | Abscess (SSTI) | ERS093084 |
| USFL326 (4) | CC8 USA300 | Abscess (SSTI) | ERS093090 |
| USFL327 (4) | CC8 USA300 | Abscess (SSTI) | ERS093091 |
| USFL330 (4) | CC8 USA300 | Abscess (SSTI) | ERS093094 |
| USFL339 (4) | CC8 USA300 | Abscess (SSTI) | ERS093103 |
| USFL341 (4) | CC8 USA300 | Abscess (SSTI) | ERS093105 |

| **NTML / Strain ID** | **Accession Number / Description** |
| --- | --- |
| LAC (AH1263) | USA300 CC8 isolate (5) |
| AH1919 | LAC complete null protease mutant (5) |
| JE2 | Wildtype strain for the NTML (6) |
| NE1 | SAUSA300_1327 |
| NE33 | SAUSA300_2589 |
| NE49 | SAUSA300_0217 (AraC family, DNA binding response regulator) |
| NE61 | SAUSA300_2150 (*lacE*, PTS system, lactose-specific IIBC component) |
| NE66 | SAUSA300_2584 |
| NE84 | SAUSA300_0741 |
| NE163 | SAUSA300_2572 (*aur,* aureolysin) |
| NE165 | SAUSA300_0114 (*sarS*, staphylococcal accessory regulator) |
| NE173 | SAUSA300_0734 (putative comf operon protein 1) |
| NE186 | SAUSA300_2441 |
| NE196 | SAUSA300_0466 (hypothetical protein) |
| NE207 | SAUSA300_1864 (*yihY*, virulence factor BrkB family protein) |
| NE226 | SAUSA300_1996 |
| NE258 | SAUSA300_2044 |
| NE264 | SAUSA300_0829 |
| NE348 | SAUSA300_1169 |
| NE370 | SAUSA300_1847 (*msbA*, hypothetical protein) |
| NE386 | SAUSA300_1708 (*rot*) |
| NE391 | SAUSA300_2565 |
| NE488 | SAUSA300_0953 (putative membrane protein) |
| NE504 | SAUSA300_1392 (phiSLT ORF191-like protein) |
| NE541 | SAUSA300_0201 (peptide ABC transporter permease protein) |
| NE543 | SAUSA300_0772 |
| NE567 | SAUSA300_2331 (*sarZ*, transcriptional regulator) |
| NE583 | SAUSA300_1716 (hypothetical protein) |
| NE643 | SAUSA300_0102 (lipoprotein) |
| NE698 | SAUSA300_1483 (hypothetical protein) |
| NE703 | SAUSA300_1750 (hypothetical protein) |
| NE707 | SAUSA300_1379 (*graD*, putative lipoprotein |
| NE727 | SAUSA300_2464 (*supH*, hydrolase, haloacid dehalogenase-like family) |
| NE810 | SAUSA300_1642 (*aapA*, alanine glycine permease) |
| NE815 | SAUSA300_0162 |
| NE839 | SAUSA300_0033 |
| NE874 | SAUSA300_1085 (*ylmH*, hypothetical protein) |
| NE934 | SAUSA300_0950 (*sspB*, cysteine protease) |
| NE1067 | SAUSA300_2266 (hypothetical protein) |
| NE1098 | SAUSA300_1756 (*splC*, serine protease) |
| NE1109 | SAUSA300_2022 (*rpoF*, RNA polymerase sigma factor SigB) |
| NE1125 | SAUSA300_0064 (*arcD*, arginine/oirnithine antiporter) |
| NE1154 | SAUSA300_0216 (*uhpT*, sugar phosphate antiporter) |
| NE1184 | SAUSA300_1328 (putative drug transporter) |
| NE1207 | SAUSA300_1757 (*splB*, serine protease) |
| NE1232 | SAUSA300_2606 (*hisF,* imidazole glycerol phosphate synthase subunit) |
| NE1247 | SAUSA300_1796 |
| NE1283 | SAUSA300_0028 |
| NE1289 | SAUSA300_0547 |
| NE1380 | SAUSA300_0978 |
| NE1439 | SAUSA300_1457 (*malR*, maltose operon transcriptional repressor) |
| NE1455 | SAUSA300_0534 (amidohydrolase) |
| NE1506 | SAUSA300_0951 (*sspA*, V8 protease) |
| NE1532 | SAUSA300_1992 (*agrA*) |
| NE1555 | SAUSA300_1148 (*codY*, transcriptional repressor) |
| NE1577 | SAUSA300_1755 (*splD*, serine protease) |
| NE1595 | SAUSA300_0588 |
| NE1602 | SAUSA300_1419 |
| NE1622 | SAUSA300_0691 (*saeR*, DNA binding response regulator) |
| NE1635 | SAUSA300_1478 |
| NE1714 | SAUSA300_1590 |
| NE1740 | SAUSA300_1445 (*scpA*, staphopain A) |
| NE1763 | SAUSA300_1480 |
| NE1764 | SAUSA300_1753 (*splF*, serine protease) |
| NE1826 | SAUSA300_1953 |
| NE1844 | SAUSA300_1754 (*splE*, serine protease) |
| NE1851 | SAUSA300_0029 (*mvaS2*, hypothetical protein) |
| NE1854 | SAUSA300_1502 |
| NE1892 | SAUSA300_1171 (hypothetical protein) |

Supplementary Table 2. Genes significantly associated with extracellular protease activity determined by GWAS

| **Gene / Unitig Name** | **hits** | **maxp** | **avg_maf** | **avg_beta** | **NTML ID** | **Locus** |
| --- | --- | --- | --- | --- | --- | --- |
| *cls_2* | 7 | 3.616 | 0.296 | 0.409 | NE258 | SAUSA300_2044 |
| NLANLCGL_01823 | 2 | 3.406 | 0.213 | 0.198 | NE196 | SAUSA300_0466 |
| NLANLCGL_02275 | 9 | 3.309 | 0.059 | 0.377 | NE698 | SAUSA300_1483 |
| FBONBOFM_00663 | 10 | 2.987 | 0.080 | 0.111 |  |  |
| FBONBOFM_00665 | 3 | 2.967 | 0.071 | 0.447 | NE1763 | SAUSA300_1480 |
| NLANLCGL_00108 | 5 | 2.790 | 0.044 | 1.177 | NE504 | SAUSA300_1392 |
| *ftsK_1* | 5 | 2.762 | 0.418 | 0.269 | NE348 | SAUSA300_1169 |
| *sdrD* | 62 | 2.742 | 0.237 | 0.370 | NE1289 | SAUSA300_0547 |
| NLANLCGL_01969 | 2 | 2.618 | 0.028 | 0.677 | NE874 | SAUSA300_1085 |
| ABAPMNGE_01918\| | 1 | 2.618 | 0.044 | 1.130 |  |  |
| IEALGAFJ_02189 | 3 | 2.270 | 0.027 | 0.660 | NE1635 | SAUSA300_1478 |
| NLANLCGL_02397 | 14 | 2.256 | 0.034 | 0.435 | NE703 | SAUSA300_1750 |
| ebh_1 | 22 | 2.167 | 0.456 | 0.581 | NE1 | SAUSA300_1327 |
| IOAEKNJG_01084 | 2 | 2.099 | 0.219 | 0.437 | NE1283 | SAUSA300_0028 |
| COBMDCKG_01388 | 1 | 2.045 | 0.444 | 0.737 | NE1851 | SAUSA300_0029 |
| ***nadE*** | **7** | **1.780** | **0.438** | **1.673** | **-** | **SAUSA300_1893** |
| *norB_4* | 4 | 1.777 | 0.500 | 0.361 | NE1184 | SAUSA300_1328 |
| *ykoD_1* | 4 | 1.777 | 0.500 | 0.387 | NE1380 | SAUSA300_0978 |
| ***sufC*** | **4** | **1.777** | **0.500** | **0.357** | **-** | **SAUSA300_0818** |
| *comFA* | 4 | 1.777 | 0.500 | 0.314 | NE173 | SAUSA300_0734 |
| ***glmS*** | **2** | **1.777** | **0.500** | **0.619** | **-** | **SAUSA300_2104** |
| *hisF* | 2 | 1.777 | 0.500 | 0.615 | NE1232 | SAUSA300_2606 |
| ***gyrB*** | **2** | **1.777** | **0.500** | **0.613** | **-** | **SAUSA300_0005** |
| *uvrB* | 2 | 1.777 | 0.500 | 0.613 | NE84 | SAUSA300_0741 |
| *secA_2* | 2 | 1.777 | 0.500 | 0.611 | NE66 | SAUSA300_2584 |
| NLANLCGL_00851 | 1 | 1.777 | 0.025 | 0.627 | NE583 | SAUSA300_1716 |
| *arcD_2* | 1 | 1.777 | 0.025 | 0.619 | NE1125 | SAUSA300_0064 |
| GPNHNPJK_00126 | 1 | 1.777 | 0.025 | 0.615 | - | SAUSA300_1891 |
| GPNHNPJK_00155 | 1 | 1.777 | 0.025 | 0.614 | NE207 | SAUSA300_1864 |
| **GPNHNPJK_00543** | **1** | **1.777** | **0.025** | **0.612** | **-** | **SAUSA300_1446** |
| *clfB* | 8 | 1.772 | 0.385 | 0.497 | NE391 | SAUSA300_2565 |
| NLANLCGL_02056 | 2 | 1.764 | 0.018 | 0.500 | NE1892 | SAUSA300_1171 |
| *uhpT* | 3 | 1.701 | 0.340 | 0.378 | NE1154 | SAUSA300_0216 |
| NLANLCGL_00122 | 5 | 1.678 | 0.041 | 0.692 | NE707 | SAUSA300_1379 |
| *clfA* | 17 | 1.656 | 0.130 | 0.835 | NE543 | SAUSA300_0772 |
| *blaR1* | 1 | 1.516 | 0.031 | 4.370 | NE839 | SAUSA300_0033 |
| NLANLCGL_00007 | 6 | 1.504 | 0.017 | 0.721 | NE643 | SAUSA300_0102 |
| *sraP* | 13 | 1.495 | 0.313 | 1.263 | NE33 | SAUSA300_2589 |
| NLANLCGL_00747 | 2 | 1.495 | 0.012 | 0.377 | NE49 | SAUSA300_0217 |
| NLANLCGL_00470 | 2 | 1.488 | 0.022 | 1.885 | NE1854 | SAUSA300_1502 |
| NLANLCGL_01629 | 4 | 1.453 | 0.015 | 0.720 | NE727 | SAUSA300_2464 |
| NLANLCGL_02564\|NLANLCGL_02565 | 2 | 1.423 | 0.022 | 0.867 | NE1067 | SAUSA300_2266 |
| NLANLCGL_02302 | 8 | 1.331 | 0.013 | 0.584 | NE1595 | SAUSA300_0588 |
| ***sufB_2*** | **4** | **1.308** | **0.256** | **0.501** | **-** | **SAUSA300_0822** |
| *sasG* | 1 | 1.308 | 0.019 | 2.070 | - | SAUSA300_2435 |
| *agrA* | 1 | 1.408 | 0.651 | 1.456 | NE1532 | SAUSA300_1992 |
| *nrgA* | 2 | 1.236 | 0.453 | 1.377 | NE226 | SAUSA300_1996 |
| *isaA* | 4 | 1.221 | 0.500 | 0.605 | - | SAUSA300_2506 |
| NLANLCGL_01455 | 4 | 1.221 | 0.500 | 0.526 | NE1455 | SAUSA300_0534 |
| ***topA*** | **4** | **1.221** | **0.500** | **0.547** | **-** | **SAUSA300_1143** |
| *hemH* | 2 | 1.221 | 0.500 | 0.966 | - | SAUSA300_1782 |
| *lacF\|lacE* | 2 | 1.221 | 0.500 | 0.967 | NE61 | SAUSA300_2150 |
| NLANLCGL_01209 | 1 | 1.221 | 0.012 | 1.020 | - | SAUSA300_2580 |
| *lipA_1* | 1 | 1.221 | 0.012 | 1.020 | NE264 | SAUSA300_0829 |
| NLANLCGL_02748 | 1 | 1.221 | 0.012 | 0.989 | NE370 | SAUSA300_1847 |
| *fnbA_1* | 5 | 1.166 | 0.405 | 0.704 | NE186 | SAUSA300_2441 |
| *malR* | 2 | 1.096 | 0.500 | 0.571 | NE1439 | SAUSA300_1457 |
| ***eno*** | **2** | **1.096** | **0.500** | **0.578** | **-** | **SAUSA300_0760** |
| NLANLCGL_01424 | 1 | 1.096 | 0.012 | 0.578 | NE541 | SAUSA300_0201 |
| *cycA_1* | 1 | 1.093 | 0.037 | 2.650 | NE810 | SAUSA300_1642 |
| **NLANLCGL_01900** | **1** | **1.092** | **0.012** | **1.140** | **-** | **SAUSA300_1022** |
| EPACMCON_01678 | 1 | 1.031 | 0.150 | 0.520 | - | SAUSA300_1643 |
| NLANLCGL_01351 | 2 | 1.006 | 0.016 | 0.770 | NE488 | SAUSA300_0953 |
| NLANLCGL_01104 | 3 | 0.896 | 0.033 | 0.684 | NE1602 | SAUSA300_1419 |
| NLANLCGL_00065 | 10 | 0.873 | 0.057 | 0.279 | NE1826 | SAUSA300_1953 |
| **INHOJILF_00006** | **1** | **0.762** | **0.013** | **2.440** | **-** | **SAUSA300_2141** |
| NLANLCGL_02482 | 1 | 0.583 | 0.012 | 1.460 | NE1247 | SAUSA300_1796 |
| NLANLCGL_00804\|NLANLCGL_00805 | 1 | 0.559 | 0.012 | 3.260 | NE815 | SAUSA300_0162 |
| ABAPMNGE_00281 | 2 | 0.426 | 0.028 | 1.679 | - | SAUSA300_1959 |
| *relA* | 1 | 0.276 | 0.012 | 7.080 | NE1714 | SAUSA300_1590 |

Hits: the number of k-mer hits found to be associated with the phenotype. Maxp: the maximum p-value for k-mers that mapped to the gene. Avg_maf: the average minor allele frequency of the proportion of isolates with the minor allele present. Ave_beta: the average effect size of variants mapping to the gene. Effect size is a measure of the predicted contribution of the variation within the gene to the observed phenotype. Genes highlighted in bold are considered essential.

**References:**

1. Recker M, Laabei M, Toleman MS, Reuter S, Saunderson RB, Blane B, et al. Clonal differences in Staphylococcus aureus bacteraemia-associated mortality. Nat Microbiol. 2017;2(10):1381-8.

2. Douglas EJA, Marshall B, Alghamadi A, Joseph EA, Duggan S, Vittorio S, et al. Improved Antibacterial Activity of 1,3,4-Oxadiazole-Based Compounds That Restrict Staphylococcus aureus Growth Independent of LtaS Function. ACS Infect Dis. 2023;9(11):2141-59.

3. Laabei M, Uhlemann AC, Lowy FD, Austin ED, Yokoyama M, Ouadi K, et al. Evolutionary Trade-Offs Underlie the Multi-faceted Virulence of Staphylococcus aureus. PLoS Biol. 2015;13(9):e1002229.

4. Uhlemann AC, Dordel J, Knox JR, Raven KE, Parkhill J, Holden MT, et al. Molecular tracing of the emergence, diversification, and transmission of S. aureus sequence type 8 in a New York community. Proc Natl Acad Sci U S A. 2014;111(18):6738-43.

5. Wormann ME, Reichmann NT, Malone CL, Horswill AR, Grundling A. Proteolytic cleavage inactivates the Staphylococcus aureus lipoteichoic acid synthase. J Bacteriol. 2011;193(19):5279-91.

6. Fey PD, Endres JL, Yajjala VK, Widhelm TJ, Boissy RJ, Bose JL, et al. A genetic resource for rapid and comprehensive phenotype screening of nonessential Staphylococcus aureus genes. mBio. 2013;4(1):e00537-12.
